# Nuclear Quantum Effects on the Dynamics of Bulk Water and Supercooled Aqueous Solutions

**DOI:** 10.1021/acs.jpclett.6c00307

**Published:** 2026-04-15

**Authors:** Jorge H. Melillo, Silvina Cerveny

**Affiliations:** † 226245 Donostia International Physics Center (DIPC), Paseo Manuel de Lardizabal 4, 20018 San Sebastián, Spain; ‡ Centro de Física de Materiales (CSIC-UPV/EHU)-Material Physics Centre (MPC), Paseo Manuel de Lardizábal 5, 20018 San Sebastián, Spain

## Abstract

Nuclear quantum effects (NQEs) strongly influence water dynamics, yet their magnitude and environmental tunability in biologically relevant systems remain poorly quantified. Using dielectric spectroscopy and calorimetry on H_2_O, D_2_O, and H_2_^18^O, we show that substituting ^16^O with ^18^O causes only a small (∼5%) mass-induced slowdown in both bulk water and supercooled solutions, confirming that H_2_O and H_2_^18^O exhibit identical quantum behavior. In bulk liquid water at 290 K, the relaxation-time ratio is ρ = τ_D2O_/τ_H2O_ ≈ 1.3, consistent with quantum simulations attributing this difference to NQEs. At supercooled temperatures (200 K), the isotope ratio ρ increases moderately to ≈2 in neutral poly­(vinyl methyl ether) solutions, and reaches ≈4 for fast-water relaxation in basic lysine solutions, revealing a 3-fold enhancement of NQEs. These findings demonstrate that biological environments can amplify the magnitude of NQEs on water dynamics, with implications for biochemistry and quantum-accurate aqueous modeling.

## Graphical abstract

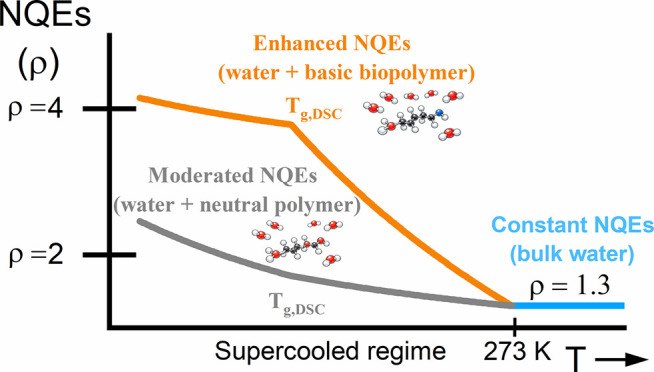

The unique properties of water (from its anomalous density maximum to its superior solvent capacity^1–7^ ) arise from a dynamic hydrogen-bond network whose quantum-mechanical nature remains not yet fully understood.^8,9^ Nuclear quantum effects (NQEs), including zero-point energy, proton delocalization, and tunneling, are known to influence the structure and dynamics of water,^10–13^ with strong differences between light water (H_2_O) and heavy water (D_2_O) at low temperatures.^14–17^ However, a critical question remains unanswered: How do biologically relevant environments (specifically charged solutes and pH) modulate these quantum contributions to water dynamics? Understanding this modulation is important because biological systems work in complex aqueous environments, where quantum effects could significantly impact function, from enzymatic catalysis to protein folding.

Disentangling nuclear quantum effects from classical mass effects has long been a challenge in water research.^12,18,19^ While H_2_O/D_2_O comparisons reveal combined mass and quantum differences,^9^ the individual contributions remain confused. Recent vibrational spectroscopy measurements revealed that charge transfer in hydrogen bonds is highly pH-dependent, with acidic solutions exhibiting more dominant NQEs than basic solutions.^20^ Whether this pH sensitivity extends to collective water reorientation and whether quantum effects can be amplified or suppressed by the solute environment remain unexplored. Here, we employ H_2_^18^O as a necessary control: this isotope differs in mass from H_2_^16^O but shares identical rotational inertia and quantum properties, enabling unambiguous separation of pure mass effects from NQEs. Using broadband dielectric spectroscopy and differential scanning calorimetry, we systematically compare H_2_O, D_2_O, and H_2_^18^O dynamics in bulk water and in supercooled aqueous solutions containing either neutral (pH = 7) poly­(vinyl methyl ether) (PVME) or basic (pH = 10.5) lysine. Supercooled conditions provide an ideal window into these effects: at low temperatures, rotational and translational motions partially decouple,^21–23^ enabling high-resolution separation of distinct relaxation processes.

Our measurements reveal a pH-dependent amplification of nuclear quantum effects. In bulk water at room temperature, D_2_O reorients ∼30% slower than H_2_O (τ_D_2_O_/τ_H_2_O_ ≈ 1.3), consistent with quantum simulations,^24^ while ^16^O→^18^O substitution produces only a ∼5% shift attributable to pure mass. We will show that, in supercooled neutral PVME solutions, the isotope ratio (ρ­(T) = τ_D_2_O_(T)/τ_H_2_O_(T), obtained from dynamical measurements (τ = (2π*f*_max_)^−1^, where *f*_max_ is determined through deconvolution of the complex loss spectra^23^) moderately increases to ≈2, but in basic lysine solutions, it reaches ≈4 (a 3-fold enhancement relative to bulk water), indicating strong NQEs within the hydration shells of water surrounding the solute. This dramatic amplification shows that biological environments can strongly enhance quantum contributions to water reorientation, with implications for understanding the role of water in biochemistry and for developing quantum-accurate models of aqueous solutions. We will also show that the ratio ρ­(T) provides a macroscopic dynamical metric of NQEs in both bulk and supercooled aqueous solutions. A similar approach was used by Chiang et al.^25^ to measure quantum effects at the air–water interface and by Gainaru et al.^15^ to analyze large isotope effects in amorphous (noncrystalline) water. Remarkably, this ratio is related to increased hydrogen bonding and the local pH environment. On the other hand, these observations above contrast with models proposing universal temperature scaling for all water properties and instead support scenarios in which NQEs become increasingly dominant as the temperature decreases, as demonstrated by Hart et al., by using viscosity measurements.^26^ Here, we use ρ­(T) as a sensitive dynamical probe to reveal how local chemistry and pH tune water reorientation in both bulk-like and hydration environments. Our results show that hydration water in vitrified, basic environments retains significant NQEs, even under confinement. These findings refine our understanding of isotope effects, quantum fluctuations, and the interplay between solvent structure and reorientational dynamics, and suggest new ways to probe the role of water in complex systems under supercooled conditions. While previous studies focused on bulk water (ρ ≈ 1.2–1.3), we demonstrate for the first time that pH can amplify this ratio to ≈4, thereby revealing unprecedented control over quantum dynamics in aqueous solutions.

The dielectric response of bulk H_2_O, D_2_O, and H_2_^18^O was investigated in the temperature range of 276–311 K, where water remains liquid. Figures 1(a,b) show the dielectric spectra of bulk H_2_^18^O, exhibiting the ε′ step and the corresponding ε″ peak. As the temperature increases, both features shift to higher frequencies, as observed in other liquids. In this frequency region (GHz), the dielectric relaxation is dominated by a single, intense process well described by a Debye function. For aqueous solutions at supercooled temperatures, the dielectric spectra become more complex due to the superposition of several relaxation processes and conductivity contributions. The relaxation times were thus determined by simultaneously fitting both the real (ε′) and imaginary (ε″) parts of the complex permittivity using Havriliak–Negami (HN) functions (eq S1 in the Supporting Information (SI)) together with a low-frequency power-law term to account for conductivity effects. To help identify the approximate positions of overlapping relaxation processes prior to fitting, the dielectric loss was reconstructed from the derivative of the real permittivity (eq S2 in SI). The relaxation times reported in this manuscript were obtained from the characteristic frequencies of the fitted HN functions. Representative fits demonstrating the quality and validity of this procedure are shown in Figure S1, and further details are provided in Analysis of Dielectric Spectra in the SI.

**Figure 1:**
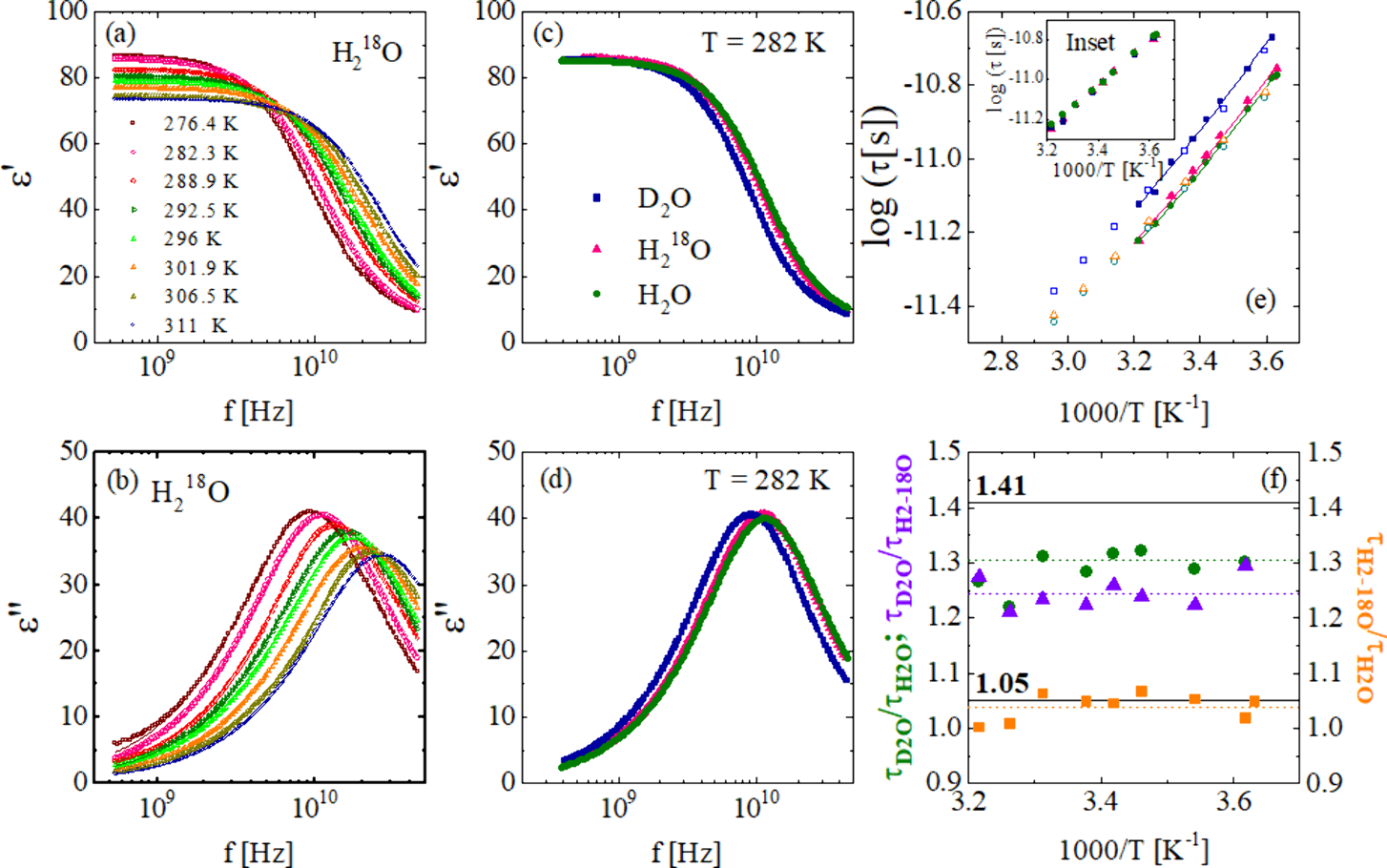
(a,b) Real and imaginary components of the complex dielectric permittivity of H_2_^18^O for temperatures between 276 and 311 K, as indicated in (a). (c,d) Comparison of the dielectric spectra for H_2_^18^O, H_2_O, and D_2_O at 282 K, revealing a slower relaxation in D_2_O relative to H_2_^18^O and H_2_O, which exhibit nearly identical dynamics. (e) Temperature-dependent relaxation for H_2_^18^O, H_2_O, and D_2_O, with VFT fits (solid lines) and literature data^9^ (open symbols). Inset: overlay of relaxation times for H_2_O, D_2_O, and H_2_^18^O after scaling by 1.31 and 1.05, respectively, for D_2_O and H_2_^18^O. (f) Isotopic ratios of relaxation times as functions of temperature; dashed lines serve as guides, solid lines denote ratios near 1.41 and 1.05.

Figures 1(c,d) compare the dielectric spectra of the three isotopic variants at 282 K, with Debye function fits shown as solid lines. The isotopic shift induced by H→D substitution is clearly visible, whereas substitution of ^16^O→^18^O produces almost no detectable change in the relaxation behavior. At the lowest temperature studied, the frequency at maximum loss is *f*_max_ ≈ 9.6 and 9.5 GHz for H_2_O and H_2_^18^O, respectively, and *f*_max_ ≈ 7.4 GHz for D_2_O, corresponding to relaxation times of τ_H_2_O_ = τ_H_2_^18^O_ ≈ 16.5 ps and τ_D_2_O_ ≈ 21.5 ps, respectively. Both H_2_O and H_2_^18^O relax significantly faster than D_2_O, consistent with the trends observed for other physical properties, such as viscosity^27^ and melting point.^28^

Figure 1(e) shows the relaxation times of the three isotopes as a function of the inverse of temperature. All three exhibit super-Arrhenius temperature dependence, well described by Vogel–Fulcher–Tammann (VFT) equation^29,30^ with strength parameter^31^ D = 5.2. Among the isotopes, H_2_O displays the fastest relaxation, closely followed by H_2_^18^O, while D_2_O is significantly slower. In particular, the relaxation times for D_2_O and H_2_^18^O can be reduced to those of H_2_O by dividing by factors of 1.31 and 1.05, respectively (see inset, Figure 1(e)). Figure 1(f) presents the ratios between the relaxation times of H_2_O, H_2_^18^O, and D_2_O, providing a quantitative measure of the isotope effect. For substitution ^16^O→^18^O, the ratio is approximately 1.05; while the H→D leads to a larger effect, τ_D_2_O_/τ_H_2_O_ ≈ 1.3. The ratio between D_2_O and H_2_^18^O is ≈1.25.

The molecular origin of the prominent loss peak in the GHz region in Figure 1(b) is widely attributed to the structural α-relaxation of water.^32,33^ If this relaxation were primarily caused by the translational motion of the entire water molecule, the isotope ratio would scale with the square root of the molecular mass, as expected from classical models of viscous transport, where characteristic times scale with the translational mass factor^27,34^ $\sqrt{m}$ :

1 $$\rho =\sqrt{\frac{m_{D_{2}O}}{m_{H_{2}O}}}=\sqrt{\frac{m_{H_{2}^{18}O}}{m_{H_{2}O}}}\approx \sqrt{\frac{20}{18}}=1.05$$

Indeed, upon oxygen substitution (^16^O→^18^O), we observe ρ ≈ 1.05, consistent with a pure mass effect. This indicates that the increased inertia of the heavier oxygen atom fully explains the slowdown in this case. In contrast, if the relaxation mechanism were solely driven by rotational diffusion around the oxygen atom, the expected isotope ratio for H→D substitution would be

2 $$\rho =\sqrt{\frac{m_{D}}{m_{H}}}\approx \sqrt{2}\approx 1.41$$

reflecting the increased moment of inertia. However, in liquid water, molecular reorientation does not proceed via free rotation. Instead, it occurs through large-angle hydrogen-bond exchange (jump) mechanisms, involving the concerted motion of water molecules and their hydrogen-bonding network.^35^ In this jump regime, the effects of inertia are diminished, yielding a smaller isotope effect than predicted by the rigid rotor model. Classical molecular dynamics simulations predict τ_D_2_O_/τ_H_2_O_ ≈ 1.11–1.15,^24^ significantly lower than the rigid-rotor limit ≈1.41. Inclusion of NQEs raises the value to τ_D_2_O_/τ_H_2_O_ ≈ 1.3, indicating that NQEs accelerate H_2_O dynamics by ≈12%, with negligible impact on D_2_O.^24^ This quantum-induced enhancement persists after correcting for mass effects: the predicted τ_D_2_O_/τ_H_2_^18^O_ ≈ 1.25 is consistent with our experimental results (see Figure 1(f)). Notably, these ratios remain essentially constant over the entire temperature range investigated, in contrast to the temperature dependence reported by Hart et al.^26^ for water above 273 K. However, for supercooled aqueous solutions, our results converge with the trend described by Hart et al., as discussed in detail below. Overall, these findings demonstrate that the GHz relaxation peak provides direct, quantitative evidence for NQEs in liquid water. At room temperature, the dominant quantum contribution arises from the zero-point energy of the O–H stretch, which modulates hydrogen bond strength and network flexibility.^12,25,36^ Ratios of dynamical and transport properties, such as diffusion or viscosity, for H_2_O and D_2_O, have been used as macroscopic measures of NQEs in water.^11,12^ Here, we show that the dielectric relaxation-time ratio ρ­(T) plays a similar role and is highly sensitive to local chemistry and pH in supercooled aqueous solutions. While previous studies have primarily focused on bulk water properties where ρ ≈ 1.2–1.3,^9,37^ our supercooled solutions reveal that this ratio can be strongly amplified by solute chemistry, reaching values as high as ≈ 4 in basic lysine solutions, as explained in detail below where we studied lysine solutions that have pH values of 10.5 in H_2_O and 10.9 in D_2_O, indicating basic conditions and PVME solutions which are pH-neutral (pH ≈ 7).

Based on previous studies of supercooled aqueous solutions, the dynamics of water have been shown to exhibit two distinct types of behavior.^38–40^ “Standard” solutions exhibit a single water relaxation process (fast-water relaxation), whereas “biolike” solutions (including proteins, amino acids, and certain polymers) exhibit both slow-water and fast-water relaxations. Fast-water relaxation (also called ν-relaxation,^41^ water relaxation^42^, or γ-relaxation^43,44^ is nearly universal in water-rich systems, following Arrhenius temperature dependence with activation energies around 0.5 eV.^41,45–47^ This process reflects bulk-like water reorientation with little solute coupling. In contrast, the slow-water relaxation occurs only in solutions where water strongly plasticizes the solute (Δ*T*_g_ ≈ 200 K between the dry and hydrated states) and reflects a cooperative dynamics between water and charged or flexible interfacial groups.^48,49^ Based on these previous studies, we discuss the dynamics of water in PVME and lysine solutions below.

Figure 2(a) displays the heat flow as a function of temperature for lysine solutions. All samples remained amorphous during both cooling and heating scans, with no sign of crystallization. The calorimetric glass transition temperature (T_g,DSC_) is nearly identical for H_2_O and H_2_^18^O, whereas D_2_O shifts T_g,DSC_ to higher temperatures. Results for PVME can be found in Figure S2 in the SI. Figure 2(b) summarizes the measured T_g,DSC_ for all systems, including previous data of tripropylene glycol (3PG) solutions.^17^ A detailed compilation of T_g,DSC_ for each solution is provided in Table S1 in the SI.

**Figure 2:**
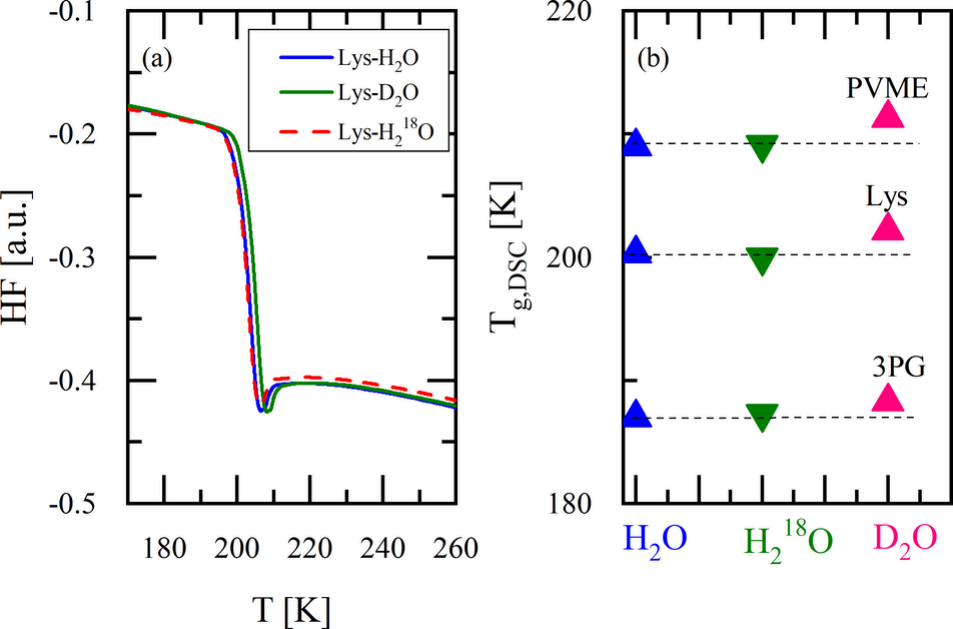
(a) DSC scans of lysine solutions prepared with H_2_O, D_2_O, and H_2_^18^O. Water content was adjusted to maintain the same number of water molecules per solute molecule across isotopes (35.0 wt % for H_2_O, 37.4 wt % for both D_2_O and H_2_^18^O). Samples were cooled to 120 K and then reheated to 290 at 10 K/min. (b) T_g,DSC_ for lysine, PVME, and 3PG solutions with all three water isotopes; 3PG data reproduced from^17^ (errors in the determination of *T*_g_ are smaller than symbols).

The dynamic response of lysine solutions was characterized by dielectric spectroscopy at temperatures between 120 and 320 K. Three relaxation processes are resolved in the spectra:^50^ a slow α-relaxation of the matrix (lysine) and two faster processes associated with slow- and fast-water dynamics, respectively. Figure 3 shows the temperature dependence of the corresponding relaxation times. For aqueous systems involving multiple relaxation processes, the uncertainty in determining the relaxation time can be up to 10%^18^ and error bars are omitted in Figure 3 to improve visual clarity. Both the α-relaxation and the slow-water relaxation follow a VFT behavior above T_g,DSC_, while below T_g,DSC_, the slow- and fast-water relaxations display Arrhenius behavior.

**Figure 3:**
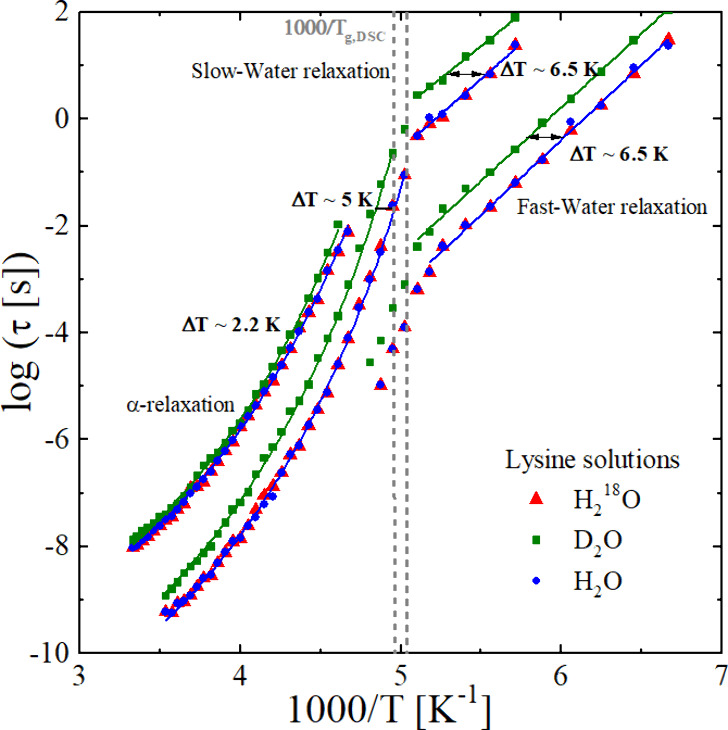
Dielectric relaxation times of lysine aqueous solutions prepared with the three water isotopes (red triangles: H_2_^18^O, green squares: D_2_O, blue circles: H_2_O) and at c_w_ = 35 wt % for H_2_O and 37.4 wt % for D_2_O and H_2_^18^O. Solid lines show VFT fits for the slow-water and fast-water and α-relaxations below 1000/T_g,DSC_, and Arrhenius fits for the slow- and fast-water relaxations above 1000/T_g,DSC_. Relaxation times for H_2_O and H_2_^18^O are nearly identical, whereas clear shifts appear when D_2_O is used as solvent. The α-relaxation exhibits a shift of 2.2 K when H_2_O and D_2_O is used as solvent, consistent with T_g,DSC_ (see. Table S1 in the SI). The slow-water relaxation shows a shift of ∼5 K just below 1000/T_g,DSC_, which decreases with increasing temperature. Above 1000/T_g,DSC_, both the slow- and fast-water relaxations display a ∼6.5 K shift.

The dielectric glass transition temperature T_g,100_ is obtained as the temperature at which the α-relaxation time extrapolates to 100 s. For lysine solutions, T_g,100_ = (196.8 ± 1.2) K for H_2_O and H_2_^18^O, and T_g,100_ = (200.8 ± 1.2) K for D_2_O, corresponding to a shift of ΔT_g,100_ = (4.0 ± 1.2) K. At supercooled temperatures, the water relaxations exhibit a larger isotopic shift than the α-relaxation. In lysine, the shift is ≈6 K for both slow- and fast-water relaxations below T_g,100_, and ≈ 5 K for the slow-water relaxation above it. In PVME, the water relaxation shifts are around 3.4 K (see Figure S3 in the SI). For comparison, the α-relaxation shows a much smaller isotope shift of about 2.2 K in lysine and roughly 1.8 K in PVME, consistent with the minimal quantum contribution to structural relaxation.

Figure 4 presents ρ­(T) for different aqueous systems at supercooled temperatures, highlighting the impact of NQEs on water dynamics under these conditions: at room temperature (Figure 1f), ρ ≈ 1.31 remains essentially constant, consistent with the lower end of the supercooled water data in Figure 4. However, upon supercooling, ρ­(T) increases dramatically, reaching values of 2–2.5 below 220 K. This strong temperature dependence reflects the growing dominance of nuclear quantum effects as thermal activation becomes less effective at lower temperatures. In previous work on 3PG-water mixtures,^17^ a composition of 15 wt % water (3PG:H_2_O ≈ 1:1.9) shows ρ­(T) ≈ 1.25 between 150 and 250 K, comparable to bulk liquid water at ambient temperature. Increasing the water content to 35 wt % raises ρ­(T) ≈ 2 at low temperatures, decreasing to ≈1.5 just above 200 K. A similar trend was found in PVME solutions in the same temperature window, where ρ­(T) varies approximately linearly with inverse temperature ρ­(T) ≈ 0.45 × (1000/T) – 0.54. In contrast, lysine solutions exhibit a comparable linear dependence for the fast-water relaxation ρ­(T) ≈ 0.22 × (1000/T) + 2.68. This shows that ρ­(T) increases with decreasing temperature, reflecting the growing influence of NQEs as thermal activation diminishes. A quantitative analysis based on the Arrhenius parameters for the two isotopes is provided in the Analysis of ρ­(T) for fast-water relaxation in the SI. This analysis shows that the approximate linear dependence of ρ­(T) on 1000/T is consistent with the small difference between the activation energies in lysine solutions. In contrast, for PVME, the exponential term cannot be accurately linearized. Nevertheless, in both cases, the linear expressions are used as a phenomenological description of the experimental trends to facilitate comparison between the different systems. A similar temperature dependence of ρ­(T) is revealed when the previously reported relaxation times for water in silica gel^19^ and bulk supercooled water^51^ are reanalyzed in terms of the isotope ratio, as shown in Figure 4.

**Figure 4:**
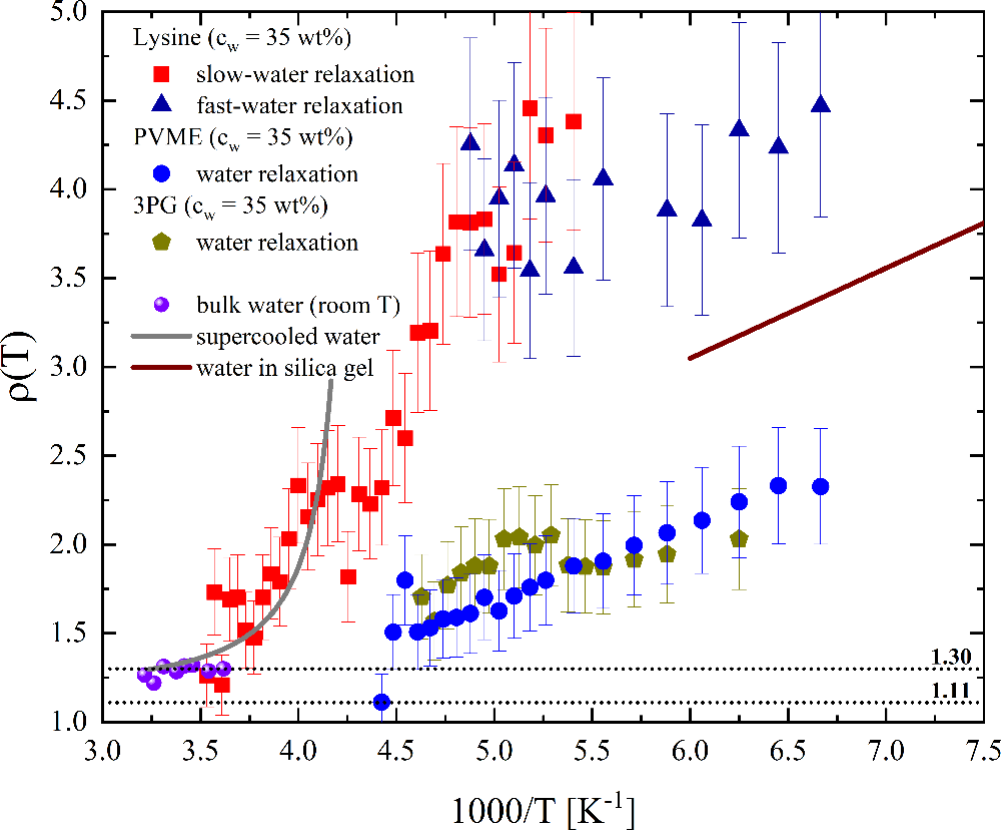
Temperature dependence of the isotope effect, ρ­(T), for dielectric relaxation in supercooled aqueous systems: lysine (35 wt %), PVME (35 wt %), 3PG (35 wt %), bulk water at room temperature (Figure 1f), and supercooled bulk water,^51^ and water in silica gel.^19^ Except for bulk water, all systems show increasing ρ­(T) with decreasing temperature, highlighting the growing influence of NQEs. Lysine solutions show the most significant isotope effects (ρ ≈ 4), whereas neutral polymers (PVME, 3PG) exhibit moderate effects. Dotted lines at ρ = 1.11 and 1.30 indicate classical and quantum simulation predictions,^24^ respectively. Errors are indicated in the Figure.

These behaviors differ from those of Kim et al.^52^ and Kringle et al.,^16^ who proposed that the dynamics of supercooled liquid D_2_O and H_2_O can be made equivalent by simple temperature scaling. To test this hypothesis, we fitted our data using the empirical relation

$$\tau_{D_{2}O,fit}\left(T\right)=\alpha \tau_{H_{2}O}\left(T+\Delta T\right)$$ 3

where ΔT represents the temperature shift associated with NQEs and α accounts for additional multiplicative corrections, such as mass-related corrections. The fitting was performed for all samples, and the results are shown in Figure S4, while the corresponding parameters are summarized in Table 1.

**Table 1:** Fitting Parameters Obtained from eq 3 for the Different Systems Studied^a^

Environment	α	ΔT [K]	R^2^
Bulk water	1.08 ± 0.7	5.09 ± 2.09	0.99
PVME	0.70 ± 0.10	5.00 ± 0.63	0.99
Lysine			
Slow-water	2.10 ± 0.25	5.00 ± 0.63	0.99
Fast-water	0.49 ± 0.02	7.73 ± 0.19	0.99

a ΔT corresponds to the NQEs-related temperature shift and α to an additional multiplicative correction.

The fits reproduce the data very well, and the extracted values of ΔT≈ 5–8 K fall within the range reported in the literature.^9,16,52^ Notably, ΔT is approximately constant across all systems, consistent with a universal quantum temperature shift. However, a significant difference emerges when examining the parameter α. For bulk water, the obtained value of α ≈ 1.08 is consistent with that reported by Kutus^9^ et al., where α corresponds to the classical mass correction of 1.05, between H_2_O and D_2_O. In this case, the isotope difference in relaxation times can be explained by a temperature shift of approximately 7 K combined with the mass correction. Moreover, for bulk water, ρ­(T) remains essentially constant with temperature. Thus, both approaches (temperature scaling and ρ­(T) analysis) consistently indicate that NQEs in bulk water are approximately temperature independent.

In contrast, for the aqueous systems investigated here (lysine and PVME aqueous solutions), the fitted values of α depend strongly on the specific system (from 0.49 to 2.10) and even on the particular water relaxation process considered, with no clear physical connection between them. These values deviate substantially from the classical mass ratio and do not follow a consistent physical pattern. For instance, in lysine solutions, α ≈ 0.49 for fast-water relaxation but α ≈ 2.10 for slow-water relaxation, even though both processes involve the same isotope pair in the same solution. This variation of α indicates that the isotope effect cannot be decomposed simply into a universal classical mass correction (α ≈ 1.05) plus a quantum temperature shift (ΔT). Instead, the coupling between water dynamics and the confining environment differs for H_2_O and D_2_O, leading to a process-dependent prefactor ratio that depends on the local hydrogen-bonding network, confinement geometry, and coupling to the matrix dynamics. Therefore, while eq 3 provides an excellent mathematical fit, the parameter α does not have a universal physical interpretation in confined systems, unlike in bulk water. Therefore, the direct analysis of ρ­(T) shown in Figure 4 provides a clear physical picture for confined water. This analysis clearly shows that, unlike bulk water where ρ ≈ 1.3 is nearly constant, ρ­(T) increases systematically from ∼ 1.5 to ∼ 5 as temperature decreases in lysine and PVME solutions, demonstrating that the magnitude of the total isotope effect is strongly temperature-dependent in these systems.

PVME and 3PG solutions have shown moderate isotope effects (ρ­(T) ≈ 2), whereas lysine solutions exhibit higher values of ρ­(T) ≈ 4. This difference probably derives from the chemical environment. Because 3PG and PVME are neutral (nonionic) polymers, they can perturb water in sosution through excluded-volume effects and hydrophobic hydration. By contrast, the amino acid lysine can create a basic environment in water (at pH ≈ 10, lysine is mostly positively charged) due to its isoelectric point (pI ≈ 9.74).^53^ The absence of strong electrostatic interactions for 3PG or PVME solutions restricts the enhancement of NQEs. At room temperature, Flór et al.^20^ reported that, in basic solutions, hydrogen bonds are strengthened while NQEs are suppressed, due to the delocalized charge of OH^–^. However, at supercooled temperatures, NQEs such as proton tunneling and delocalization are expected to become more pronounced,^12^ particularly in the presence of strong hydrogen bonds.^13^ Shorter O···O distances reduce the width of the barrier, facilitating tunneling and delocalization.^54^ This mechanism may underlie the pronounced isotope effects in lysine solutions, where strengthened hydrogen bonding at low temperature amplifies NQEs and accelerates H_2_O relaxation relative to D_2_O. Even larger values, ρ­(T) ≈ 10, were reported by Heres et al.^55^ for phosphoric acid, attributed to efficient proton transfer processes driven by zero-point energy differences. Acidic systems generally exhibit stronger NQEs than basic ones at room temperature,^20^ and it is plausible that NQEs are further amplified in acid environments under supercooled conditions. Table 2 summarizes the investigated water environments, their isotope ratios, and the associated qualitative remarks.

**Table 2:** Approximate Isotope Relaxation-Time Ratios ρ­(T) = τ_D2O_/τ_H2O_ for Water in Different Environments^a^

Environment	ρ(T)	Comment
Bulk liquid water (room T)	∼1.3	GHz dielectric relaxation; NQEs enhance H_2_O dynamics
Supercooled bulk water^51^	1.3–3	moderate NQEs; strongly increases on cooling
3PG solution (15 wt % H_2_O)^17^	up to ∼1.3	Strong confinement; weak NQEs
3PG solution (35 wt % H_2_O)^17^	up to ∼2	More bulk-like water; moderate enhancement
PVME solution (35 wt % H_2_O)	up to ∼2	Neutral polymer; moderate NQEs
Lysine solution (35 wt % H_2_O)	up to ∼4	Basic environment; strongly enhanced NQEs
Phosphoric acid^55^	up to ∼10	Proton-transfer dominated and very strong isotope effect

a Values refer to the low-temperature supercooled regime, when applicable.

While nuclear quantum effects are the main cause of the large isotope effects seen in our study (ρ ≈ 4 in lysine, up to ρ ≈ 10 in acidic systems), a full understanding requires quantitative decomposition of the contributions. Using our three-isotope method, we can separate: (i) Classical mass effects, which contribute a constant baseline factor of ρ ≈ 1.05, demonstrated by oxygen substitution (H_2_^18^O/H_2_O). This accounts for about 2% of the overall slowdown in lysine at 210 K. (ii) Nuclear quantum effects (zero-point energy and tunneling) provide the main contribution with an amplification factor of ρ­(D_2_O/H_2_O)/ρ­(H_2_^18^O/H_2_O) = 4/1.05 ≈ 3.8 in lysine at 210 K. This represents approximately 98% of the enhancement. (iii) Environment and temperature effects: at similar temperatures (∼220 K), the quantum amplification factor ranges from ∼ 1.4 in bulk water to ∼ 3.8 in basic lysine and up to ∼ 9.5 in acidic phosphoric acid, reflecting different hydrogen-bonding networks. Considering a single system, the quantum factor increases when the temperature decreases (see Figure 4). While environment and temperature affect the observed isotope effects, they do so mainly by adjusting the size of nuclear quantum effects rather than adding separate contributions. Therefore, the three-isotope method allows this quantitative separation, showing that about 98% of the enhancement in lysine stems from quantum effects, whose size is influenced by local chemistry (pH, hydrogen bonding) and temperature.

Finally, for the slow-water relaxation below T_g,DSC_, the isotope ratio ρ­(T) matches that of the fast-water relaxation, indicating a shared quantum-limited mechanism in the vitrified regime. Above T_g,DSC_, the fast-water relaxation leaves the measurement window, and ρ­(T) for the slow-water (such as 3PG and PVME) exhibits moderate quantum effects. In contrast, basic solutes such as lysine produce stronger NQEs, although still smaller than those reported for acidic solutions. The isotope effect ρ­(T) for the water relaxation decreases with increasing temperature, consistent with thermal suppression of NQEs. At higher temperatures, thermal energy dominates molecular motion, reducing the relative importance of quantum zero-point energy and tunneling contributions. Below T_g,DSC_, water is confined within the matrix, yet proton tunneling and delocalization persist, leading to a slow but finite temperature dependence of ρ­(T). The slow relaxation is typically attributed to hydration water,^49,50^ which in lysine solutions forms a shell around the charged −NH_3_^+^ and −COO^–^ groups. Solutions for which water is strongly confined (as in 3PG at 15 wt %) show negligible NQEs.^17^ In contrast, lysine solutions maintain sufficient hydrogen-bond flexibility to support quantum fluctuations. Simulations and dielectric measurements indicate that cooperative hydration shells around lysine extend up to two solvation layers^56^ and remain dynamically active even under confinement. Furthermore, neutron diffraction experiments and computational simulations indicate that the hydration environments retain their fundamental water-like characteristics,^57^ thus preserving the necessary conditions to tune nuclear quantum effects and quantify their influence on water dynamics. The marked isotope effects observed in lysine solutions therefore emerge from a delicate balance between electrostatic confinement imposed by the solute and the cooperative quantum dynamics operating within the hydration shell. This observation leads to an important conclusion: the magnitude of nuclear quantum effects in water should not be regarded as a universal constant. Instead, it represents a tunable property that can be modulated through solute chemistry and pH adjustment. This tunability carries important implications for a wide range of supercooled aqueous systems, including biomolecular hydration layers and cryopreservation applications.

In conclusion, we have demonstrated that nuclear quantum effects in water are not universal constants, but tunable properties controlled by pH and solute chemistry. Using H_2_^18^O as a mass-effect control, we show that the isotope ratio ρ­(T) increases from ∼1.3 in bulk water to ∼4 in basic lysine solutions at supercooled temperatures. These findings have important implications for understanding water in biological systems, where charged amino acids and pH gradients are always present. The 3-fold enhancement of NQEs in lysine solutions suggests that quantum effects may be significantly larger in protein hydration shells than in bulk water. This could affect enzyme catalysis, protein folding, and other processes where water dynamics play a key role. Additionally, the pH-tunability of NQEs opens possibilities for controlling water dynamics in technological applications such as cryopreservation, where the stability of hydrogen-bond networks and water mobility are key factors governing ice formation and biomolecular preservation. In these applications, the ability to modulate ice formation kinetics and the dynamics of water in the unfrozen fraction could improve cell viability during freezing and thawing processes. Since nuclear quantum effects significantly influence hydrogen bonding and thermodynamic properties of water, they may also play a role in determining the microscopic dynamics of water in cryoprotective environments.^58,59^

## Experimental Section

Aqueous solutions of PVME, 3PG, and lysine were prepared using three different water types: H_2_O, D_2_O, or H_2_^18^O (all chemicals from Sigma-Aldrich; see details in the SI). The water content of each mixture is adjusted so that all three isotopic solutions contain the same number of water molecules per solute molecule, as summarized in Table S1 of the SI. This ensures a uniform hydration level across isotopes (c_w_ = 35 wt % for H_2_O, 37.4 wt % for H_2_^18^O, and 37.4 wt % for D_2_O). The sample preparation includes purifying the solutes by ion exchange, lyophilizing, and mixing with the isotopic water in a glovebox; the samples were sealed for several months to ensure homogeneous water distribution. Differential scanning calorimetry (DSC) was used to determine glass transition temperatures (T_g,DSC_) and to analyze the amorphous states under the dielectric conditions. The complex dielectric permittivity, ε*­(ω) = ε′(ω) – iε″(ω), was measured over the frequency range 10^–1^ to 10^6^ Hz using a Novocontrol Alpha Analyzer. The sample thickness for all measurements was 0.1 mm, and the sample diameter was 30 mm. In the GHz region (10^6^ to 10^10^ Hz), dielectric spectra were obtained using a Hewlett-Packard (HP) HP-85070E dielectric probe kit with an open-ended coaxial probe connected to a vector network analyzer (VNA) HP-8361 was used. VNA was calibrated using air, water, and a short circuit as calibration standards. The liquids were prepared in a cylindrical glass container with a 20 mm diameter. Full experimental details are provided in the SI (Experimental Details).

## Supplementary Material



## Notes

Experimental details of the samples, descriptions of the DSC and BDS techniques, a detailed account of the dielectric spectra analysis, the analysis of ρ­(T) for fast-water relaxation, test of empirical temperature-shift scaling for H_2_O and D_2_O relaxation times, and DSC and BDS results for PVME solutions (DOCX)
