# Supplementary material for: Nuclear Quantum Effects on the Dynamics of Bulk Water and Supercooled Aqueous Solutions
Source: J Phys Chem Lett. 2026 Apr 15;17(17):4906–13. doi: 10.1021/acs.jpclett.6c00307 (PMC13137237; doi:10.1021/acs.jpclett.6c00307)
Supplement: Supplementary file 1 [file jz6c00307_si_001.docx]

**Supporting Information for “Nuclear Quantum Effects on the Dynamics of Bulk Water and Supercooled Aqueous Solutions”**

Jorge H. Melillo^1^, Silvina Cerveny^2*^

^1^Donostia International Physics Center (DIPC), Paseo Manuel de Lardizabal 4 (20018), San Sebastián, Spain.

^2^Centro de Física de Materiales (CSIC-UPV/EHU)-Material Physics Centre (MPC), Paseo Manuel de Lardizábal 5 (20018), San Sebastián, Spain.

**Experimental Details**

Aqueous solutions of PVME, 3PG, and lysine were prepared using three different water isotopes: H_2_O, D_2_O, and H_2_^18^O. H_2_O (product 95284), D_2_O (product 151882), and H_2_^18^O (product 329878) were purchased from Sigma-Aldrich. PVME (product 182729) and lysine (M_w_ = 146.19 g mol-1, product L5501) were obtained from Aldrich Chemical. The AG 501-X8 mixed-bed ion-exchange resin used for purification was purchased from Bio-Rad Laboratories.

The water content of each mixture was adjusted so that all three isotopic solutions contained the same number of water molecules per solute molecule, ensuring uniform hydration across isotopes (c_w_ = 35 wt% for H_2_O, 37.4 wt% for H_2_^18^O, and 37.4 wt% for D_2_O). Sample preparation involved solute purification by ion exchange, lyophilization, and subsequent mixing with isotopic water inside a glovebox. The samples were then sealed and stored for several months to ensure homogeneous water distribution. The final water contents are summarized in Table S1.

**Table S1.** General data of solutions at a concentration of 35 wt%. N corresponds to the number of water molecules per solute molecule, T_g,DSC_ denotes the glass transition temperature measured by calorimetry at 10 K/min and ΔT_g_ represents the shift in glass transition temperature between solutions prepared in H_2_O or H_2_^18^O and those in D_2_O.

| Solute | Solvent | N | T_g,DSC_ [K] | ΔT_g_ = T_g,H2O_ – T_g,D2O_ [K] |
| --- | --- | --- | --- | --- |
| Lysine | H_2_O | 4.44 | 200.3 | 2.2 ± 0.2 |
|  | H_2_^18^O | 4.44 | 200.0 |  |
|  | D_2_O | 4.43 | 202.2 |  |
| PVME | H_2_O | 657 | 209.0 | 1.3 ± 0.3 |
|  | H_2_^18^O | 657 | 209.1 |  |
|  | D_2_O | 654 | 211.3 |  |
| 3PG | H_2_O | 5.75 | 188.1 | 1.3 ± 0.3 |
|  | H_2_^18^O | 5.75 | 188.2 |  |
|  | D_2_O | 5.75 | 189.4 |  |

Calorimetric measurements described in the manuscript were performed using a DSC Q2000 from TA Instruments in standard mode. Samples weighing about 10-15 mg were prepared in hermetic pans inside a globebox. DSC experiments were performed at heating and cooling rates of 5 and 10 K/min as indicated in each experiment. A helium flow rate of 25 mL/min was used throughout. T_g,DSC_ was obtained from the onset of the glass transition step in the heat flow curve. Table S1 reports the T_g,DSC_ values for each solution, along with the difference between the T_g,DSC_ using D_2_O and H_2_O as solvents.

The complex dielectric permittivity, ε*(ω) = ε′(ω) - i ε″(ω), was measured over the frequency range 10^-1^ to 10^6^ Hz using a Novocontrol Alpha Analyzer. The sample thickness for all measurements was 0.1 mm, and the sample diameter was 30 mm. In the GHz region (10^6^ to 10^10^ Hz), dielectric spectra were obtained using a Hewlett-Packard (HP) HP-85070E dielectric probe kit with an open-ended coaxial probe connected to a vector network analyzer (VNA) HP-8361 was used. VNA was calibrated using air, water, and a short circuit as calibration standards. The liquids were prepared in a cylindrical glass container with a 20 mm diameter.

**Analysis of Dielectric Spectra**

The complex permittivity can be fitted using the phenomenological Havriliak-Negami (HN) function

$\varepsilon^{*}\left( \omega\right)={\varepsilon^{'}\left( \omega\right)+i\varepsilon^{''}\left( \omega\right)=\varepsilon}_{\infty}+\frac{\Delta\varepsilon}{\left[ 1+\left( i\omega\tau\right)^{\alpha} \right]^{\beta}}$ (S1)

where ∆ε is the dielectric strength, *ε_∞_* the unrelaxed value of the dielectric constant, τ is the relaxation time and *ω* = 2πf is the angular frequency. In eq. 1, *α* and *β* are shape parameters (0 < α, αβ ≤ 1) which describe the symmetric and the asymmetric broadening of the equivalent relaxation time distribution function. By setting *α* = *β* = 1 a Debye function is obtained, whereas setting *β* = 1 a symmetrical function is obtained (Cole-Cole (CC) function), which is widely used to describe secondary relaxations in glassy materials.

At low frequencies, conductivity effects dominate, and to account for that, a power law term was added to the sum of CC and/or HN functions. To analyze the complex permittivity without the contribution from these conductivity effects, ε′′ can be approximated from the derivative of ε′

${\varepsilon^{''}}_{der}\left( \omega\right) \approx-\frac{\pi}{2} \frac{\partial\varepsilon^{'}(\omega)}{\partial(log(\omega))}$ (S2)

Figures S1 (a,b) show spectra at temperatures below T_g,DSC_, where both the fast- and slow-water relaxations are resolved, while panel (c) shows a temperature above T_g,DSC_ where the α-relaxation becomes visible. The results demonstrate that applying Eq. (2) is essential for a correct analysis; without it, the relaxation peaks cannot be reliably distinguished.

*
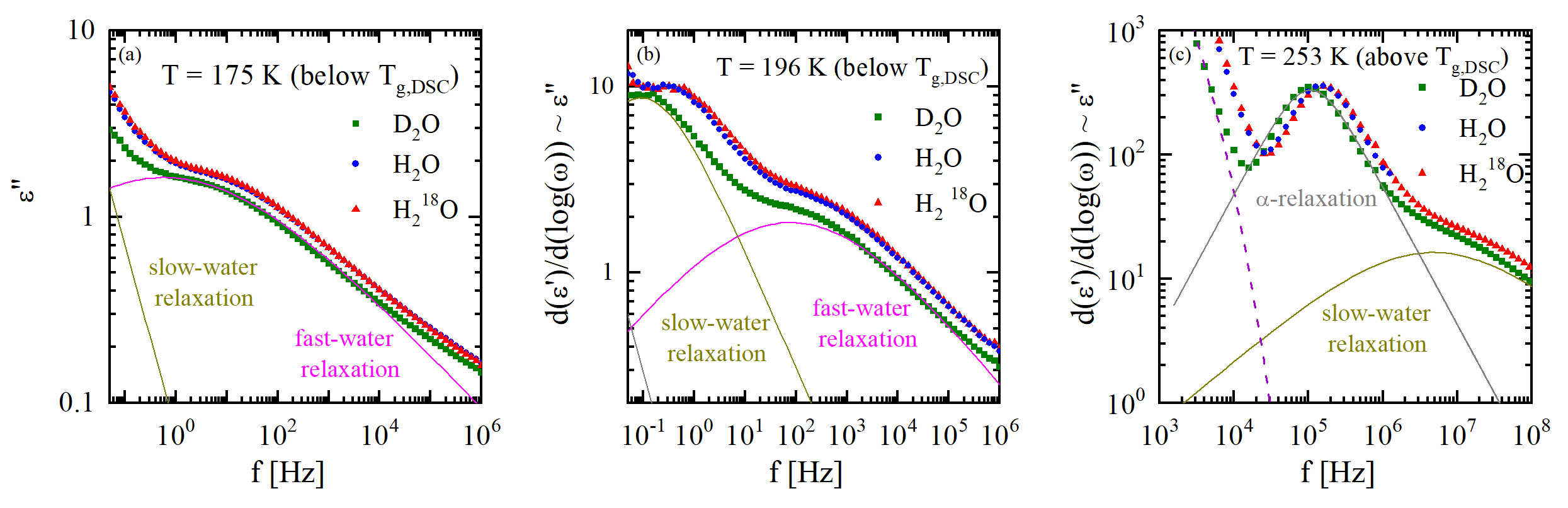
*

**Figure S1.** Imaginary part of the dielectric permittivity for lysine solutions prepared with D_2_O (green squares), H_2_O (blue circles), and H_2_^18^O (red triangles). (a,b) Dielectric spectra at temperatures below T_g,DSC_, where both slow-water and fast-water relaxations are resolved. (c) Spectrum at a temperature above T_g,DSC_, showing the emergence of the α-relaxation. The spectra were reconstructed using Eq. 2, which obtains ε″ from the derivative of ε′, allowing a reliable separation of the overlapping relaxation processes. The relaxation processes shown correspond to the Cole-Cole function fits performed for the D_2_O solution; fits for the other isotopic solvents yield similar processes but are omitted for clarity.

The relaxation times exhibit distinct temperature dependences above and below T_g,DSC_. Below T_g,DSC_, the fast-water relaxation follows an Arrhenius behavior (Eq. 3). In contrast, above T_g,DSC_, the structural α-relaxation is well described by a Vogel-Fulcher-Tammann (VFT) law (Eq. 4). The dielectric glass-transition temperature, T_g,100,_ was defined as the temperature at which the VFT extrapolated α-relaxation time satisfies $\tau_{\alpha}\left( T_{g,100} \right)=100$ s.

$\tau(T)= \tau_{0} e^{\frac{E_{a}}{k_{b}T}}$ (S3)

$\tau(T)= \tau_{0} e^{\frac{D T_{0}}{T- T_{0}}}$ (S4)

**Analysis of ρ(T) for fast-water relaxation**

To analyze the temperature dependence of the isotope ratio ρ(T) for the fast-water relaxation below T_g,DSC_, we start from the Arrhenius expression describing the relaxation time (Eq. 3 in this Supporting Information). The isotope ratio between the relaxation times of D_2_O and H_2_O is therefore:

$\rho\left( T \right)=\frac{\tau_{D2O}\left( T \right)}{\tau_{H2O}\left( T \right)}= \frac{\tau_{0,D2O} e^{\frac{E_{a,D2O}}{k_{b}T}}}{\tau_{0,H2O}e^{\frac{E_{a,H2O}}{k_{b}T}}}= \frac{\tau_{0.D2O}}{\tau_{0,H2O}} e^{\frac{E_{a,D2O}- E_{a,H2O}}{k_{b}T}}$ (S5)

When the activation energies of the two isotopes are similar and $=\frac{E_{a,D_{2}O}-E_{a,H_{2}O}}{k_{B}T}\approx0$ , the exponential term can be approximated by a first-order Taylor expansion $\exp\left( x \right)\approx1+x$ with $x=(E_{a,D_{2}O}-E_{a,H_{2}O})/(k_{B}T)$. Under this approximation, Eq. (5) becomes

$\rho\left( T \right)=\frac{\tau_{0,D2O}}{\tau_{0,H2O}}\left( 1+\frac{E_{a,D2O}- E_{a,H2O}}{k_{b}T} \right)$ (S6)

which can be rewritten as

$\rho\left( T \right)= \frac{\tau_{0,D2O}}{\tau_{0,H2O}}+ \frac{\tau_{0,D2O} (E_{a,D2O}- E_{a,H2O})}{1000 \tau_{0,H2O} k_{b}} \left( \frac{1000}{T} \right)$ (S7)

Thus, under the assumption of similar activation energies, ρ(T) is expected to show a linear dependence on 1000/T. The validity of this approximation can be evaluated through the expansion parameter $x$. Considering a representative temperature of T = 200 K, for lysine solutions we obtain $x\approx0.24$. In this case, the first-order approximation is valid, and the observed linear dependence of ρ(T) on 1000/T can be rationalized directly from the Arrhenius parameters.

In contrast, for PVME solutions, the parameter is significantly larger ($x\approx1.98$), indicating that a first-order expansion cannot accurately approximate the exponential term. However, we retain the linear representation as a phenomenological description of the experimental trend, which facilitates direct comparison with the lysine system.

**Calorimetry and Dynamics Results of PVME Solutions**

Figure S2 displays the heat flow as a function of temperature for PVME solutions using H_2_^18^O and D_2_O as solvents. Upon cooling at 10 K/min, crystallization is fully suppressed, whereas partial crystallization appears during the subsequent heating scan. Nevertheless, the glass transition remains clearly identifiable, and in the temperature range where the samples remain amorphous, they can be reliably studied by dielectric spectroscopy.


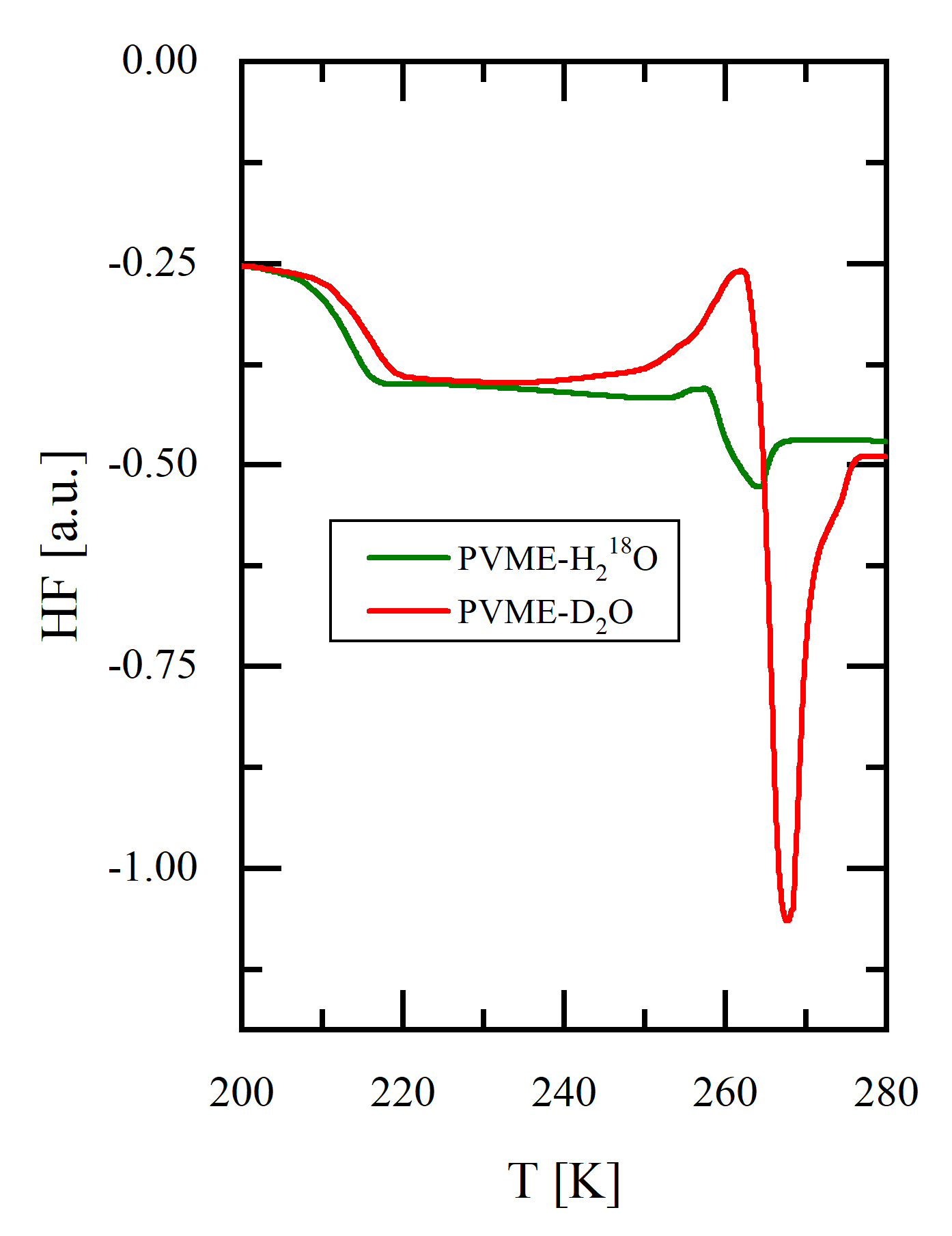


**Figure S2.** Heat flow curves of PVME solutions prepared with H₂¹⁸O and D₂O as solvents. Cooling at 10 K/min suppresses crystallization, while partial crystallization occurs during the subsequent heating scan.

Figure S3 shows the temperature dependence of the relaxation times for PVME aqueous solutions in the temperature range where the samples remain amorphous. PVME solutions behave as conventional aqueous mixtures, exhibiting two relaxation processes: the α-relaxation, which follows a VFT temperature dependence, and the fast-water relaxation, which follows an Arrhenius law below T_g,DSC_. The fast-water relaxation displays an isotope-induced shift of approximately 3.4 K between solutions prepared with D_2_O and H_2_O.


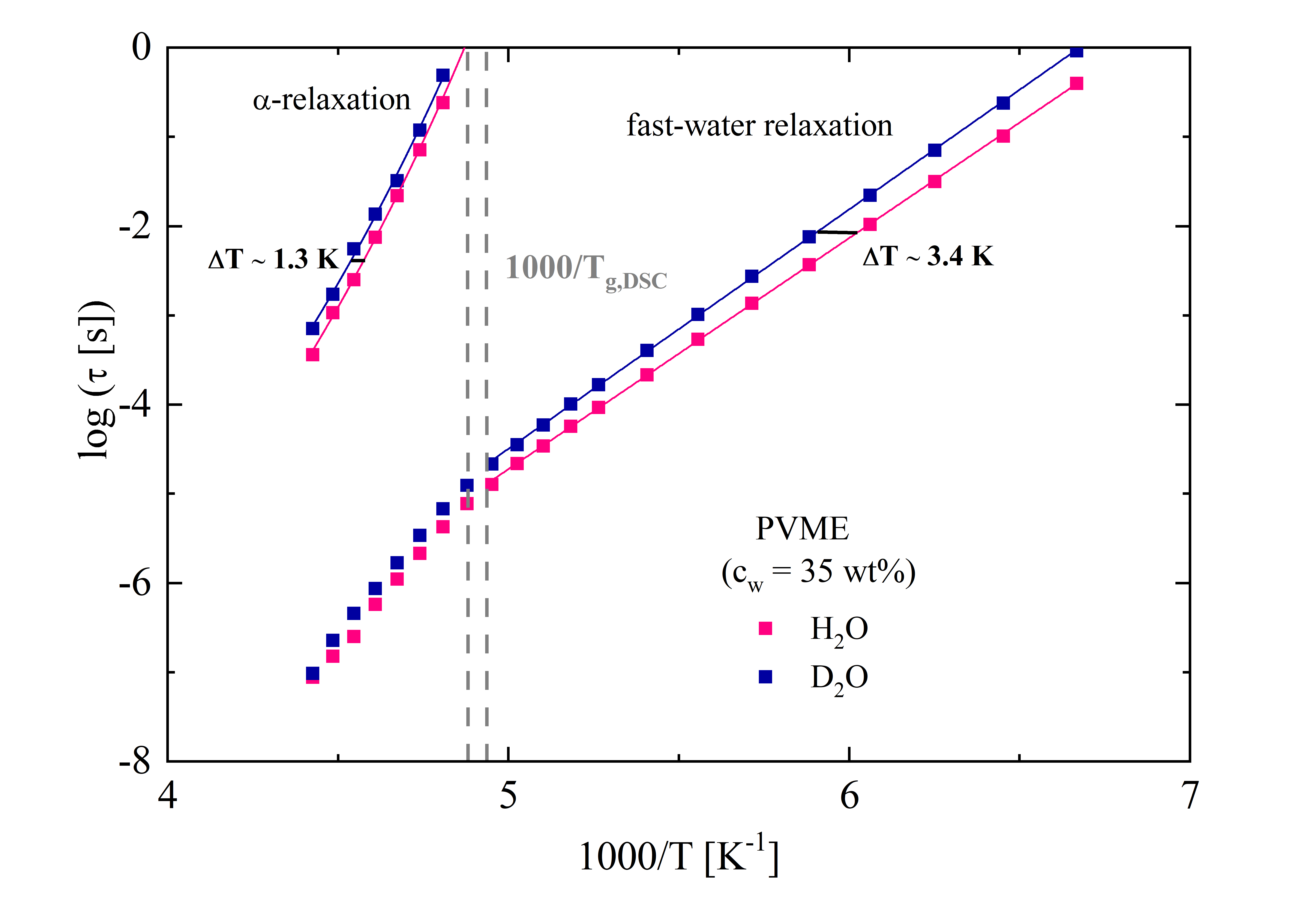


**Figure S3.** Dielectric relaxation times of PVME aqueous solutions prepared with the H_2_O and D_2_O as solvents. Above 1000/T_g,DSC_, fast-water relaxations display a ~ 3.4 K shift.

**Test of empirical temperature-shift scaling for H_2_O and D_2_O relaxation times**

Figure S4 shows the results of fitting the empirical relation of Eq. 3 in the manuscript ($\tau_{D_{2}O,fit}(T)=\alpha\text{ }\tau_{H_{2}O}(T+\Delta T)$). Where ΔT represents a temperature shift associated with nuclear quantum effects and α is an additional multiplicative factor often interpreted as a classical mass correction. The resulting fits reproduce the experimental D_2_O relaxation times for all systems investigated in this work. The extracted parameters are summarized in Table 1 in the manuscript. While the values of ΔT fall within the range reported in the literature, the parameter α varies significantly across systems and relaxation processes and lacks a systematic physical interpretation. These results indicate that although the empirical scaling reproduces the relaxation times mathematically, it does not provide a consistent physical description across the different aqueous environments studied here.

**
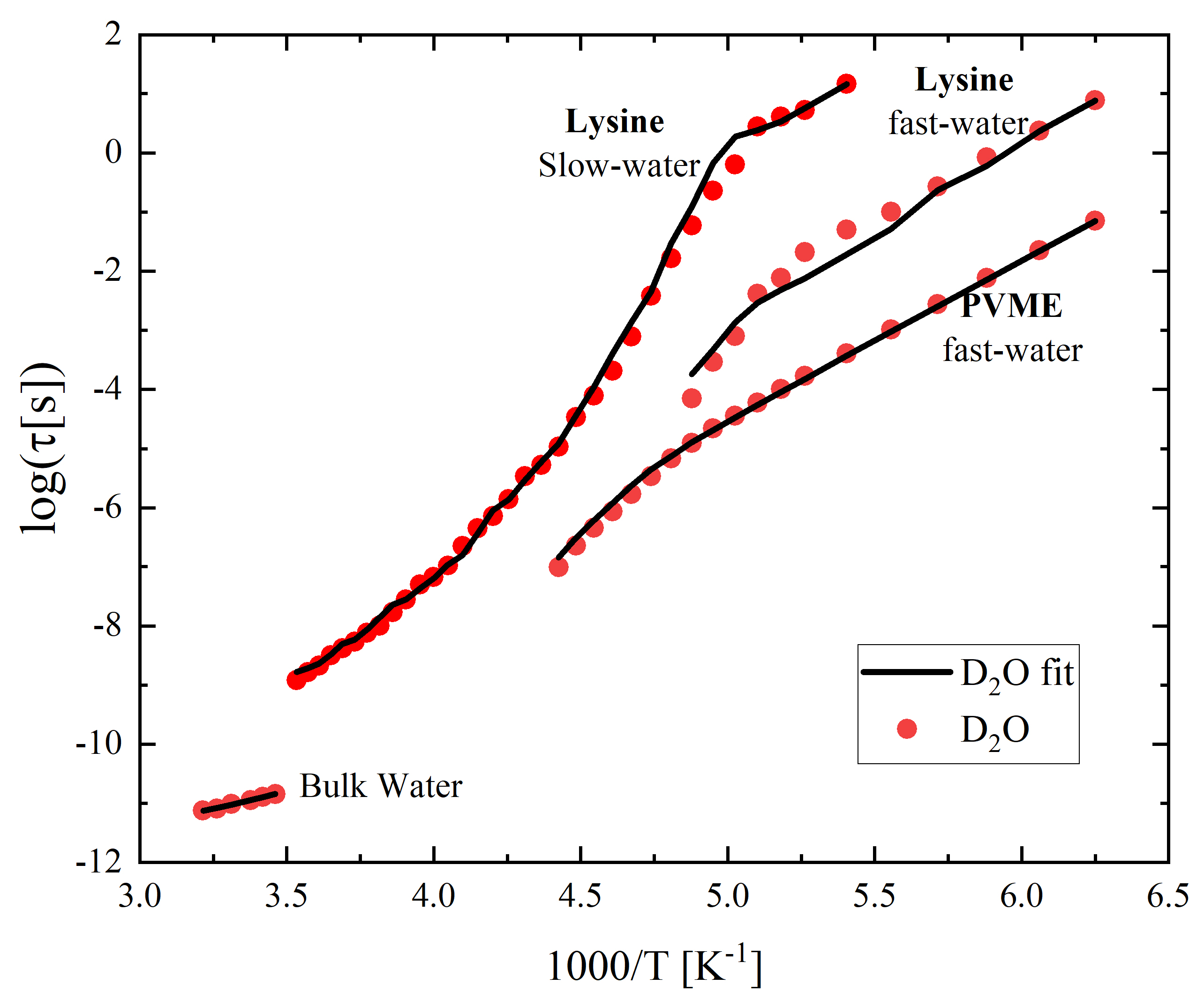
**

**Figure S4.** Comparison between the experimental relaxation times of D_2_O (red circles) and the values obtained from the empirical scaling relation of Eq. 3 from the manuscript (Black squares). The scaling parameters α and ΔT were obtained by fitting the H_2_O relaxation times for each system. The scaling reproduces the temperature dependence of the D_2_O relaxation times for bulk water and the aqueous solutions studied here, including lysine (slow- and fast-water relaxations) and PVME (fast-water relaxation). The fitting parameters are reported in Table 1 in the manuscript.
